# Electrophysiological Responses of Eighteen Species of Insects to Fire Ant Alarm Pheromone

**DOI:** 10.3390/insects10110403

**Published:** 2019-11-14

**Authors:** Yuzhe Du, Michael J. Grodowitz, Jian Chen

**Affiliations:** National Biological Control Laboratory, Biological Control of Pests Research Unit, Agriculture Research Service, United States Department of Agriculture, 59 Lee Road, Stoneville, MS 38776, USA; yuzhe.du@ars.usda.gov (Y.D.); michael.grodowitz@ars.usda.gov (M.J.G.)

**Keywords:** Alarm pheromone, *Solenopsis invicta*, EAG, GC-EAD, semiochemical

## Abstract

Olfaction plays a dominant role in insect communication. Alarm pheromones, which alert other insects of the same species of impending danger, are a major class of releaser pheromones. The major components of alarm pheromones in red imported fire ants, honeybees and aphids have been identified as 2-ethyl-3,6-dimethylpyrazine (2E-3,6-DP), isopentyl acetate (IPA), and E-β-farnesene (EβF), respectively. In this study, electroantennography (EAG) responses to EDP (a mixture of 2-ethyl-3,6-dimethylpyrazine and 2-ethyl-3,5-dimethylpyrazine), IPA and EβF were investigated in a wide range of insect species. Beside imported fire ants, the EDP (2-ethyl-3,6(5)-dimethylpyrazine) elicited significant EAG response from all other tested insects, including six ant species and one hybrid ant, honeybee, bagrada bug, lady beetle, housefly, small hive beetle, yellow fever mosquito, termite, bedbug, water hyacinth weevil, southern green stink bug and two aphid species. In contrast, IPA elicited significant EAG response only in the honeybee, red imported fire ant, an *Aphaenogaster* ant, and the water hyacinth weevil. The EβF only elicited EAG responses in two aphids, small hive beetle and housefly. The results clearly indicate that EDP can be detected by widespread insect species that did not coevolve with *S. invicta* and further suggested alkylpyrazine may activate multiple generally tuned olfactory receptors (ORs) across a wide number of insect species.

## 1. Introduction

The red imported fire ant, *Solenopsis invicta* Buren (Hymenoptera: Formicidae), is one of the worst invasive alien species in the world [1]. *S. invicta* was introduced from South America into the United States in Mobile, Alabama in the 1930s. Since then, it has spread extensively and become well established in 15 states in the U.S. [2]. Fire ants are a significant public health hazard and an important pest in agriculture with an annual loss estimated to surpass $7 billion due to damage repair and control costs [3].

Like all other social insects, *S. invicta,* depend on sophisticated pheromonal communication for maintaining colony cohesiveness, sociality, and defense. Several pheromones have been identified in *S. invicta*, including trail following, queen recognition and alarm pheromones [4,5,6,7]. An alkylpyrazine compound released by the mandibular gland, 2-ethyl-3,6-dimethylpyrazine, has been identified as the primary component of the alarm pheromone in *S. invicta* [6]. The alarm pheromone can induce *S. invicta* to express a variety of behaviors, including rapid running, attraction to the source, colony dispersal, and aggressive postures [8]. Several other alkylpyrazine analogs can elicit electroantennogram (EAG) response and alarm behaviors of *S. invicta* and 2-ethyl-3,5-dimethylpyrazine, an isomer of the pheromone, does so with nearly the same effectiveness as 2-ethyl-3,6-dimethylpyrazine [9].

Extensive research has also been conducted on alarm pheromones of honeybee and aphids. In the early 1960s, two main alarm pheromones were defined in the workers of the honeybee, *Apis mellifera* Linnaeus [10,11]. These included isopentyl acetate (IPA), the major component in the sting alarm pheromone released by the Koschevnikov gland [10], and the mandibular gland pheromone containing 2-heptanone [11]. Both substances elicit defensive behavior against intruders at the hive entrance [10,11]. In the 1970s, E-β-farnesene (EβF) was confirmed as the key alarm pheromone for many aphid species [12,13]. It is released from the cornicles, a pair of specialized structures located dorsally on the 5th or 6th abdominal segments. The response of aphids to EβF is species-dependent [14,15] and context-dependent [16,17]. Typical behaviors in response to EβF include cessation of feeding, moving away from the signal source, and often dropping from the host plant [18].

Defense is critical to ant colony survival, therefore, alarm signals generally evoke rapid and robust behavioral responses [19,20]. Although alarm pheromones may belong to many different types of molecules, such as terpenoids, alcohols, aldehydes, ketones, esters, nitrogen heterocycles, and sulfur-containing compounds [21], in general, they are small molecules with high volatility. Alarm pheromones are considered to be the least specific insect pheromone [6]. When fire ant, honeybee and aphids release alarm pheromones for defense, the alarm signals may be detected by other insect species. For example, *Apis cerana* Fabricius foragers avoid the distinctive alarm pheromones of *Apis dorsata* Fabricius and *A. mellifera*, species that share the same floral resources and predators [22]. Phorid flies in the genus *Pseudacteon* are highly specific parasitoids to red imported fire ants. Phorid flies apparently use fire ant alarm pheromone to locate their hosts [23]. Many aphid natural enemies use aphid alarm pheromones (EβF) to find their hosts [24]. Even mammals may detect and respond to insect alarm pheromones. For example, African bush elephants have been shown to respond to a honeybee alarm pheromone blend and express avoidance behaviors [25]. Therefore, knowledge of how alarm pheromones affect other species in natural environments will enhance our understanding of such interspecific interactions.

Insects can detect a large number of volatile compounds using various morphologically distinct olfactory sensilla [26,27]. Such sensilla are most commonly found associated with the antenna, though they have also been identified on the maxillary palps or proboscis, wings, and other bodily surfaces. Sensilla morphologies often relate to what odors a sensillum can detect most effectively, however, the odors detected are ultimately determined by the olfactory receptors (ORs). ORs are expressed on the dendritic membranes of olfactory sensory neurons (OSNs) suspended in an aqueous sensillar lymph [27]. Formation of a heteromeric complex is required in insect chemoreception, which requires at least one OR and a co-expressed OR (Orco) [28,29]. Interestingly, while Orco [30] is highly conserved among insects, the sequences of other OR genes exhibit very little sequence similarity even within the same insect order [31]. However, ORs being broadly tuned, able to respond to wide variety of molecules and the ratio of responses creates the odor signal [32,33]. This ability to detect a broad number of odors often allows insects to detect odorants they did not coevolve with. The EAG technique, which measures the electric potential between two ends of the antenna, has been used to study olfactory mechanisms in insects for several decades. The EAG represents the summation of bioelectrical potentials generated by many antennal ORs responding almost simultaneously [34].

Red imported fire ants are effective predators of insects, however, little or no information is available on whether other insects can detect fire ant alarm pheromone and aid in avoiding predation. Such information is important in understanding the interaction of fire ants and their prey. Discrimination of honeybee and aphid alarm pheromones by other insects have been reported in a few cases though more research is warranted. In this study, we use EAG as a probe to investigate the electrophysiological response to alarm pheromones of red imported fire ants, honeybees and aphids in a wide range of insect species to assess the potential olfactory discrimination of these well-defined alarm pheromones by other insects.

## 2. Methods and Materials

### 2.1. Insects

Eighteen species of insects and one hybrid ant, belonging to five orders and 11 families, were used in this study (Table 1). *Solenopsis invicta* colonies were maintained in trays coated using Fluon (BioQuip Products, Rancho Dominguez, CA, USA) and kept in insect rearing rooms at 26 °C. The social form of *S. invicta* colonies was determined using PCR on Gp-9 alleles [35]. All *S. invicta* ants used in this study were from monogyne colonies. Colonies were fed with 10% sucrose and frozen house crickets, *Acheta domesticus* L. at ~70% humidity and 16:8 dark: light photoperiod. Black imported fire ants, *Solenopsis richteri* Forel, hybrid imported fire ants, *S. invicta ×S. richteri*, aphenogaster ants, *Aphaenogaster picea* Wheeler, pharaoh ants, *Monomorium pharaonic* Linnaeus, and little black ant, *Monomorium minimum* Buckley were maintained in insect rearing rooms using the same environmental conditions. Nine-spotted lady beetle, *Coccinella novemnotata* Herbst, houseflies, *Musca domestica* Linnaeus and yellow fever mosquitoes, *Aedes aegypti* Linnaeus were kept in insect rearing rooms with the same humidity and light condition. Small hive beetle, *Aethina tumida* Murray, southern green stink bug, *Nezara viridula* Linnaeus, eastern subterranean termite, *Reticulitermes flavipes* Kollar, water hyacinth weevil, *Neochetina eichhorniae* Warner, and oleander aphid, *Aphis nerii* Boyer de Foscolombe were field-collected in Mississippi (see the detail in Table 1). Honeybee, *Apis mellifera* Linnaeus was obtained from colonies maintained by the Southern Insect Management Research Unit (SIMRU), USDA-ARS, Stoneville, MS. Bird cherry-oat aphid, *Rhopalosiphum padi* Linnaeus was obtained from laboratory colonies maintained at the Biological Control of Pests Research Unit (BCPRU), USDA-ARS, Stoneville, MS. Tawny crazy ant, *Nylanderia fulva* Mayr were collected from Jackson County, MS and bagrada bug, *Bagrada hilaris* Burmeister was obtained from colonies maintained at the USDA-ARS, the BCPRU, Stoneville, Mississippi since 2012. The original individuals used to establish this colony were obtained near the University of California (Riverside, CA, USA) from London rocket (*Sisymbrium irio* L.) and shortpod mustard (*Hirschfeldia incana* (L.) Lagr.-Foss.) in the fall of 2010. The bed bug, *Cimex lectularius* Linnaeus, was obtained from Dr. Changlu Wang, Rutgers University, New Brunswick, NJ and kept at room temperature (−~25 °C).

### 2.2. Chemicals

Red imported fire ant alarm pheromone 2-ethyl-3,5(6)-dimethyl pyrazine (EDP) (a mixture of the 3,5- and 3,6-dimethyl isomers) with 90% purity was purchased from Sigma-Aldrich (Sigma-Aldrich, St. Louis, MO, USA). Honeybee alarm pheromone component, isopentyl acetate (IPA), and aphid alarm pheromone, E-β-farnesene (EβF) were purchased from Sigma-Aldrich (St. Louis, MO, USA) as well. The purities for IPA and EβF were ≥90% and ≥95%, respectively.

### 2.3. Electrophysiological Recordings

Electroantennography was performed to determine the antennal responses of 18 insect species and one hybrid ant to EDP and EβF, and eight species to IPA. Most insects were not anesthetized prior to antenna excision, except for houseflies, *M. domestica*, yellow fever mosquitoes, *A.aegypti* and honeybee, *A. mellifera*. EAG were recorded using saline-filled capillary glass electrodes [9]. EAG responses of *S. invicta* to its own alarm pheromone (EDP) were determined for major workers and male and female alates. Only workers were used for the following social insects: *S. richteri*, *S. invicta × S. richteri*, *A. picea*, *M. pharaonis*, *M. minimum*, *N. fulva*, *A. mellifera*, and *R. flavipes*. Males and females were evaluated for the non-social insects including *B. hilaris*, *C. novemnotata*, *M. domestica*, *A. aegypti*, *A. tumida*, *N. viridula*, *N. eichhorniae*, and *C. lectularius*. Only females were evaluated for the two aphid species, *A. merii* and *R. padi*. Procedures for the EAG on a majority of the species tested were similar to those used for *S. invicta* with the exception of *R. flavips*, *M. pharaonis*, *M. minimum*, *M. domestica*, *R. padi,* and *A. nerii* because of their small size and/or morphology of the antennae.

For EAG, silver wires in two glass capillary (1.1 mm in diameter) electrodes filled with saline solutions served as the reference and recording electrodes. The antenna was excised using fine-tipped forceps. The base and tip of the antenna were connected to the reference electrode and the recording electrode based on procedures modified after Kaissling and Thorson (1980) [36]. Due to the small size of the antennae in *R. flavipes*, *M. pharaonis*, *M. minimum*, *R. padi,* and *A. nerii*, the reference electrode was connected to the isolated head, while the recording electrode was connected to the tip of the antenna. Since the housefly has aristate antenna, the reference electrode was inserted to the isolated head and the recording electrode was connected to the third antennal segment.

To test the EAG response, each pheromone was dissolved in pentane and a 10 μL aliquot of the solution was then applied to a Whatman filter paper strip (3 mm × 40 mm). The pentane was allowed to evaporate by gently shaking for 10 s under a fume hood. Then the strip was inserted into the glass Pasteur pipette (Fisher Scientific, Pittsburgh, PA, USA), and the tip and the end of the pipette were immediately sealed with parafilm. Filter paper strips treated only with pentane in a glass pipette served as the control. The tip of the pipette was fitted into a side port of an L-shaped glass tube (130 mm in length and × 12 mm in diameter) oriented 5 mm away from the antennal preparation. The antenna was exposed to a solvent control blank (10 μL pentane) at the start and the end of recordings for each antenna. The stimuli were provided as 0.5 s puffs of air into a continuous humidified air stream as generated by an air stimulus controller (CS-55, Syntech^®^, Buchenbach, Baden-Württemberg, The Netherlands, Europe). EAG signals were recorded for 10 s, starting 1 s before the onset of the stimulus pulse. At least 1 min was allowed between each puff for the recovery of antennal receptors. The analog signal was detected through a probe (INR-II, Syntech^®^), captured, and processed with a data acquisition controller (IDAC-4, Syntech^®^), and later analyzed using EAGPro computer software (Syntech^®^ ).

In order to select an adequate concentration of EDP for measuring EAG response of other insects, a dose–response relationship between EDP and *S. invicta* was established. Four concentrations of EDP were used, including 0.1, 1, 10, 100 ug/μL dissolved in pentane. A linear relationship was found between 100 μg/μL and 0.1 μg/μL, and 100 μg/μL elicited the maximum EAG responses in *S. invicta.* Therefore, 100 μg/μL EDP was used for all EAG measurements for other insect species. For IPA and EβF, 100 μg/μL was also used for all the insect species.

### 2.4. GC-EAD Analysis on Commercial 2-ethyl-3,6(5)-dimethylpyrazine

In order to confirm *S. invicta*’s EAG response to the commercial EDP product (a mixture of 2-ethyl-3,6-dimethylpyrazine and 2-ethyl-3,5-dimethylpyrazine), gas chromatography–electroantennographic detection (GC-EAD) analyses were performed with *S. invicta* workers. The antennal preparation was the same as described for EAG previously.

We used an Agilent7890B GC (Agilent Technologies, Santa Clara, CA, USA) with a flame ionization detector (FID) equipped with an HP-5 MS capillary column (30 m × 0.25 mm ID × 0.25 μm film thickness, Agilent) with nitrogen as the carrier. The oven temperature was held at 50 °C for 2 min, increased to 240 °C at 10 °C/min, and then held at this temperature for 4 min. The column effluent was split 1:1 for simultaneous detection by both detectors (FID and EAD). The transfer tube to the EAD preparation was heated to 230 °C and the outlet for the EAD was delivered to the insect antenna through an L-shaped glass tube (12 cm × 6 mm I.D.) in a humidified airstream. 1 μL EDP at 10 μg/μL was injected, EAD and FID signals were captured and processed with GC/EAD 2000 software (Syntech, Co., Ltd., Tianjin, China).

### 2.5. Data Analysis

All EAG responses were recorded in mV. For establishing a dose–response relationship of EAG response to EDP for *S. invicta*, EAG recordings were obtained from 6–12 antennal preparations from each of three colonies for each dose. Results are reported as mean ± SEM (standard error of the mean). EAG response to the pentane control (average of two recordings per antennal preparation) was deducted from the EAG amplitudes. For each of the tested insect species, 6–12 antennal was prepared and its EAG responses to EDP, IPA, and EβF at 100 μg/μL were compared to their responses to pentane. For each compound, EAG responses were obtained from antennal preparations. Statistical significance in EAG response among doses for each caste of *S. invicta*, among castes for each dose, among compounds for each insect species or among species for each compound was determined using one-way ANOVA followed by Tukey’s test, with the significance threshold set at *p* < 0.05 (OriginLab Corporation, Northampton, MA, USA). A positive EAG response was defined if a compound elicited EAG signals with an average amplitude significantly higher than that of the solvent control using ANOVA followed by Tukey’s test.

## 3. Results

### 3.1. Gas Chromatography-Electroantennographic Detection (GC-EAD) Analysis of S. invicta to 2-ethyl-3,5(6)-dimethyl pyrazine (EDP)

**Figure 1:**
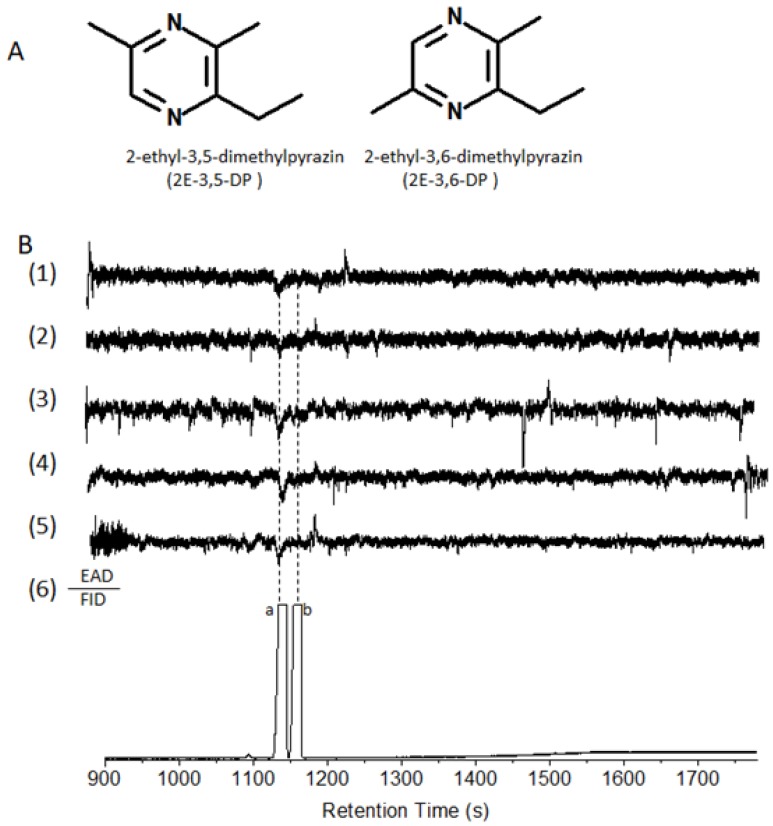
GC-EAD responses of *S. invicta* workers, female and male alates to the alarm pheromone 2-ethyl-3,6-dimethylpyrazine (2E-3,6,-DP) and its isomer 2-ethyl-3,5-dimethylpyrazine (2E-3,5,-DP). (**A**) Chemical structures of 2E-3,5,-DP and 2E-3,6,-DP. (**B**) EAD responses of female alates (1, 5), male alates (2) and workers (3, 4) and FID chromatogram (6) (peak a: 2E-3,6-DP and peak b: 2E-3,5-DP).

The commercially available 2-ethyl-3,5(6)-dimethyl pyrazine mainly contains 2-ethyl-3,5-dimethyl pyrazine and 2-ethyl-3,6-dimethyl pyrazine (Figure 1A) were detected by FID (Figure 1B (6)). The 2-ethyl-3,6-dimethylpyrazine reported as fire ant alarm pheromone elicited electroantennographic detection (EAD) response in *S. invicta* females (Figure 1B (1) and (5)), males (Figure 1B (2)) alate and workers (Figure 1B (3) and (4)). However, 2-ethyl-3,5-dimethylpyrazine could not be detected in several antennae preparations (Figure 1B (4) and (5)). Overall, GC-EAD signals were generally very small for 2-ethyl-3,6-dimethylpyrazine. Since pure fire ant alarm pheromone was not available, we originally thought that GC-EAD might be useful to measure the response of other insects to fire ant alarm pheromone in the isomer mixture. However, since the GC-EAD response was too small to meet the need in this study, we elected to use the isomer mixture EDP with direct EAG measurement.

### 3.2. Concentration Dependent EAG Responses to EDP in S. invicta Workers, Male, and Female Alates

**Figure 2:**
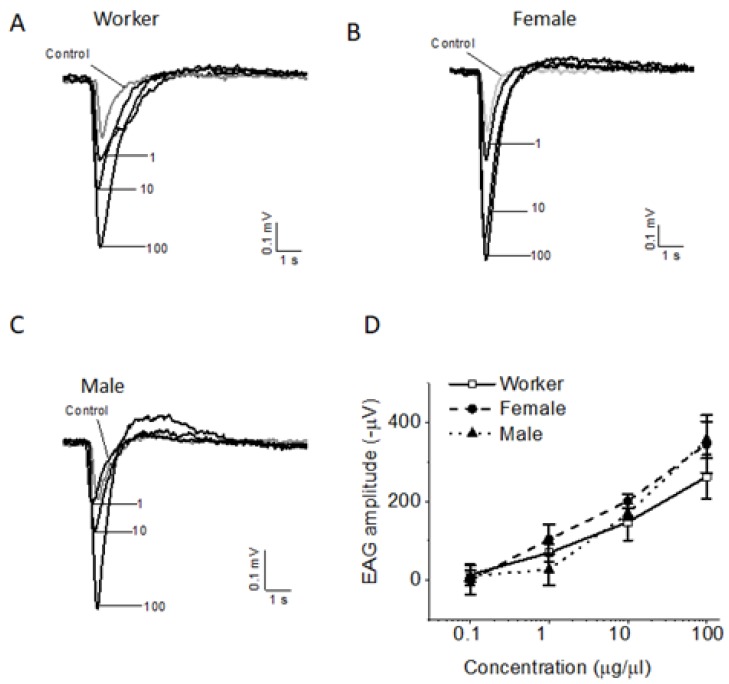
Fire ant alarm pheromone, EDP, elicited concentration-dependent EAG response in *S. invicta* workers (**A**), female alates (**B**) and male alates (**C**). Dose-response curves (**D**) for workers, male and female alates. Each point represents the mean (±SEM) of six to ten tested antennae (**D**).

EAG responses of *S. invicta* workers, female and male alates to EDP were measured at four doses, 0.1, 1, 10, 100 μg/μL. EDP elicited clear concentration-dependent EAG-responses in workers (Figure 2A) and both female (Figure 2B) and male alates (Figure 2C). A positive correlation was found between EAG responses and the doses of EDP. The *r*-value (correlation coefficient) was 0.9, 0.87, and 0.93 for workers (n = 7), female alates (n = 6) and male alates (n = 8), respectively. The EAG amplitude reached 0.26 ± 0.05 mV, 0.35 ± 0.07 mV and 0.36 ± 0.05 mV at 100 μg/μL for workers or female and male alates, when pentane control signal was subtracted from original EAG amplitude (Figure 2D). There was no significant difference in EAG response among workers, as well as female and male alates (*p* > 0.05).

### 3.3. EAG Response of Other Ants to Red Imported Fire Ant, Honeybee and Aphid Alarm Pheromones

**Figure 3:**
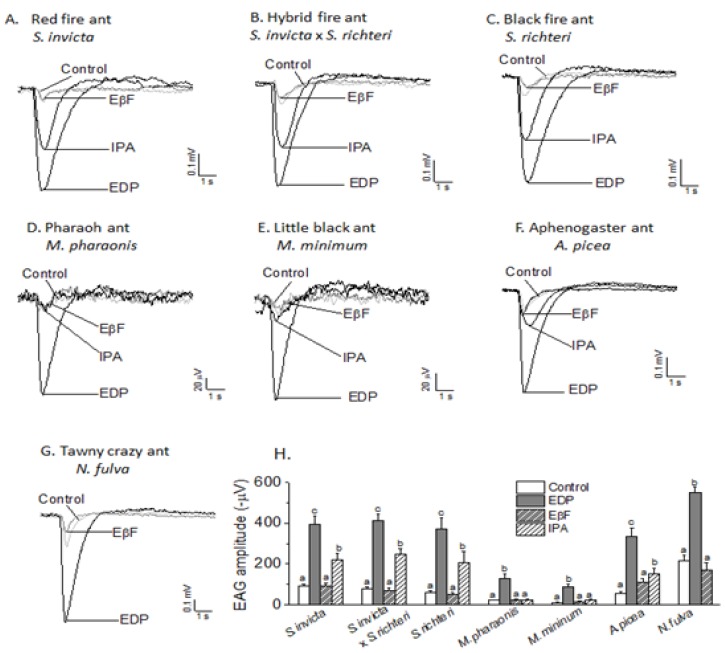
The representative EAG traces elicited by 100 μg/μL EDP, EβF and IPA in *S. invicta* (**A**), hybrid fire ant (*S. invicta* x *S. richteri*) (**B**), black imported fire ant (*S. richteri*) (**C**), pharaoh ant (*M. pharaonis*) (D), little black ant (*M. minimum*) (E), aphenogaster ant (*A. picea)*(F), and tawny crazy ant (*N. fulva*) (G) The histogram (**H**) shows the EAG responses in different ants species (mean ± SEM). Means sharing no letter on the top of bars are significantly different, as determined by one-way analysis of variance with Tukey’s test, and significant values were set at *p* < 0.05. The ANOVA was performed for each individual species response to the odorants and the differing letters only have meaning within a species, not between all species.

In addition to *S. invicta*, we examined EAG responses of *S. richteri*, *S. invicta* x *S. richteri*, *A. picea*, *M. pharaonis*, *M. minimum* and *N. fulva* to EDP, IPA and EβF. Only workers were used in all tests. EDP elicited EAG response in *S. richteri* and *S. invicta* x *S. richteri* (Figure 3A–C), *M. pharaonic* and *M. minimum* (Figure 3D–E), *A. picea* (Figure 3F) and *N. fulva,* (Figure 3G). IPA elicited EAG response in *S. invicta*, *S. richteri*, *S. invicta* x *S. richteri* and *A. picea*, however, all responses were significantly lower than their responses to EDP (*p* < 0.05), which were only about one-half of the amplitude of EDP. In addition, IPA didn’t elicit EAG response in *M. pharaonic* and *M. minimum* and EβF did not elicit any EAG responses in all six ant species (Figure 3G).

### 3.4. EAG Response of Other Insects to Red Imported Fire Ant, Honeybee and Aphid Alarm Pheromones.

**Figure 4:**
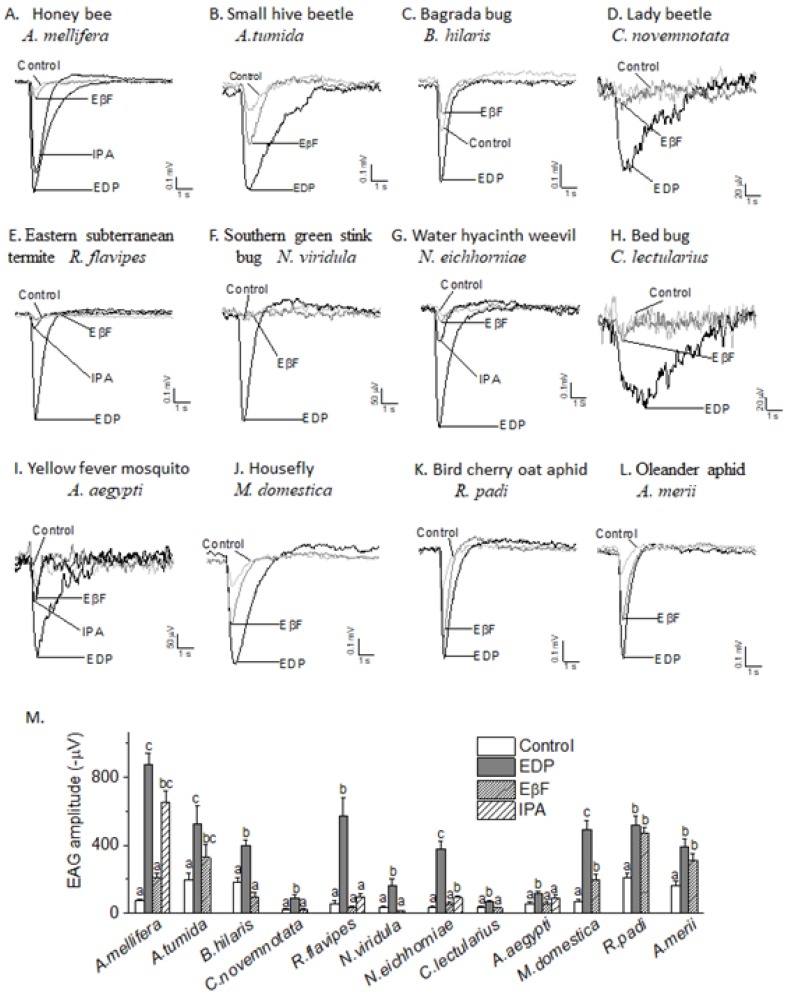
The representative EAG traces elicited by 100 μg/μL EDP, EβF and IPA dilution in honeybee (*A. mellifera*) (**A**), small hive beetle (*A.tumida*) (**B**), begrada bug (*B. hilaris*) (**C**), nine-spotted lady beetle (*C. novemnotata*) (**D**), eastern subterranean termite (*R. flavipes*) (**E**), southern green stink bug *(N. viridula*) (**F**), water hyacinth weevil (*N. eichhorniae*) (**G**), bed bug (*C. lectularius*) (**H**), yellow fever mosquito (*A. aegypti*) (**I**), housefly (*M. domestica*) (**J**), bird cherry oat aphid (*R. padi*) (**K**) and oleander aphid (*A. merii*) (**L**). M. the histogram (**M**) shows the EAG responses (mean ± SEM) in all 12 insect species. The sample for each insect species consisted of 6–12 antennae. Means sharing no letter on the top of bars are significantly different, as determined by one-way ANOVA with Tukey’s test, and significant values were set at *p* < 0.05. The ANOVA was performed for each individual species response to the odorants and the differing letters only have meaning within a species, not between all species.

As shown in Figure 4A–L, EDP elicited EAG responses in all 12 insect species tested (Figure 4M) (*p* < 0.05), including workers of *A. mellifera* (Figure 4A) and *R. flavipes* (Figure 4E). Moreover, EDP also elicited EAG responses in *A. tumida* (Figure 4B), *B. hilaris* (Figure 4C), *C. novemnotata* (Figure 4D), *N. viridula* (Figure 4F), *N. eichhorniae* (Figure 4G), *C. lectularius* (Figure 4H), *A. aegypti* (Figure 4I), *M. domestica* (Figure 4J) and two aphids species *R. padi* (Figure 4K) and *A. merii* (Figure 4L) (data on male not shown) (*p* < 0.05).

Honeybee alarm pheromone IPA elicited EAG responses in honeybee *A. mellifera* (Figure 4A) and aphid alarm pheromone EβF also elicited EAG responses in two aphid species, *R. padi* (Figure 4K) and *A. merii* (Figure 4L). Their EAG amplitudes were not significantly different from their EAG responses to EDP (*p* > 0.05) (Figure 4M). Besides honeybee *A. mellifera,* we also tested three other species for their EAG response to IPA. As a result, IPA elicited insignificant EAG response in *R. flavipes* (Figure 4E) *and A. aegypti* (Figure 4I), but significant responses in *N. eichhorniae* (Figure 4G). EβF were tested in 12 insect species. Except *A. tumida* (Figure 4B) and *M. domestica* (Figure 4J)*,* EβF did not elicit EAG responses in any other insect species (Figure 4M).

As shown in Figure 4A–L, probably due to a quite different antenna morphology, EAG amplitudes induced by alarm pheromones were quite different among insect species. EDP elicited the highest EAG responses in *A. mellifera*, and the lowest in *C. novemnotata* and *C. lectularius* (Figure 4M). EAG amplitude reached over 900 μV in *A. mellifera* worker (Figure 4A), and only about 100 μV in *C. novemnotata* (Figure 4D) and *C. lectularius* (Figure 4H). However, irrespective of the size of the EAG peak (amplitude), those EAG responses were all statistically significant to EDP (*p* < 0.05).

## 4. Discussion

Our results may provide useful information on the heterospecific effect of *S. invicta* alarm pheromone. As one of the most aggressive ants, *S. invicta* is an effective predator and includes many insects in its diet [37,38]. Sensing the predator’s alarm pheromone has an advantage for prey since it can help prey detect the presence of the predators faster and consequently improve their survival by having more time to escape. Natural selection often favors organisms with ability to rapidly and acutely sense their enemies. Sensing fire ant alarm pheromone by other insects has been demonstrated in their interaction with phorid flies, parasitoids of fire ant workers [39]. In this study, all 18 insect species from 5 different orders exhibited EAG response to EDP. Unlike *Pseudacteon*, these insects are not sympatric to *S. invicta* since it was introduced to North America in 1930s. This is strong evidence that many insects that did not coevolve with *S. invicta* can detect fire ant alarm pheromone. It is not possible for these insects evolve the ability to detect *S. invicta* alarm pheromone in just under a century. The most likely explanation is that alkylpyrazine compounds are not only confined to *S. invicta*. For example, EDP has been identified as the alarm pheromone of several other ant species in the genus of *Solenopsis* [40], and alkylpyrazines have also been found in other ant species, such as *Wasmannia auropunctata* [41] and *Aphaenogaster rudis* [42] in the subfamily Myrmicinae, *Myrmecia gulosa* [43], in the subfamily Myrmiciinae, *Calomyrmex splendidus* [44] and *Notoncus ectatommoide* [45] in subfamily Formicinae, and *Linepithema humile* [46], *I. purpurreus* [47], *I. nitidus, I. rufoniger*, and *Dolichoderus clarkia* [48] in subfamily Dolichoderinae. Alkylpyrazines are also commonly seen in the subfamily Ponerinae. Among 33 analyzed species of ponerine ants, alkylpyrazines were found in 24 species [49]. Ants are generalist predators and “are arguably the greatest success story in the history of terrestrial metazoa” [50]. Ants thrive in most ecosystems and may form 15–25% of the terrestrial animal biomass [50]. Alkylpyrazines were also found in other insects besides ants, including *Triatoma dimidiate* Latreille (Hemiptera: Reduviidae) which feeds on the blood of a wide variety of animals [51]. Interestingly, alkylpyrazines were also found to be kairomones in wolf urine and induce avoidance and fear-related behaviors in deer and mice [52,53,54]. In the course of evolution, predatory pressure from ants and other animals that use EDP or alkylpyrazine analogs as alarm pheromones may be common for many insects.

The ability of an animal to detect, discriminate, and respond to odors depends on the function of its OSNs, which in turn depends ultimately on ORs [55]. The OSN dendrites express OR proteins, which function as odor-gated ion channels in insects [56]. There were at least 60 receptors in the well-studied adult fruit fly, *Drosophila melanogaster*, with additional chemosensory receptors in the larva [57]. ORs can be narrowly tuned to certain odorants, but are often more broadly tuned, able to respond to a wide variety of odorants [32,33]. Insect chemosensory systems contain both narrowly and broadly tuned receptors, providing the basis for both specified dedicated channels for certain compounds and combinatorial coding for others as EDP [33]. In *Anopheles gambiae*, a family of 79 AgORs were cloned and expressed in South African clawed frog, *Xenopus lavis,* oocytes and most AgORs responded strongly to heterocyclic or aromatic compounds that contain a benzene ring, molecules close in structure to EDP. Some odorants, especially aromatic and heterocyclic compounds, elicited responses from multiple receptors, and other odorants strongly activated only a single AgOR [58]. *S. invicta* has 333 SiOrs identified [59]. EDP elicited strong and significant EAG response in *S.invicta* and all other 17 insect species tested, which means these insect species can detect EDP, and further indicated that many insect species might bear homology to *S. invicta* ORs, which can be activated by fire ant alarm pheromone. Although the sequences of ORs genes exhibit very little sequence similarity among insect orders [25], ORs for EDP may be highly conserved across different insect orders. Or, EDP may activate multiple more generally tuned ORs across a widespread number of insect species. These may provide useful information to others seeking to identify the responsive ORs in the future.

This study demonstrated for the first time that EβF elicited significant EAG response in housefly and small hive beetle in addition to aphids. Since both insects are important pests, it is worth defining their behavioral responses to EβF. It was demonstrated that ApisOR5, as well as two A. pisum odorant binding proteins (OBPs, ApisOBP3 and ApisOBP7), bind to EβF in *A. pisum* [60]. EDP elicited strong EAG in two tested aphids. Whether EDP can also bind to the same ORs and OBPs as EβF does in *R. padi* and *A. merii* is an interesting topic for future research.

Alarm pheromones from predators may be a rich source of general insect repellants. Insects are major agricultural pests and their control largely depends on synthetic insecticides. Such dependence on insecticides has caused the development of insecticide resistance, environmental pollution and negative impact on non-target organisms. Metabolic engineering of crops for resistance to pest insects using a non-toxic mode of action is a potential alternative strategy. For example, engineering crops to have ability to synthesize and emit EβF could reduce aphid infestations by repelling aphids and attracting their natural enemies [61]. Although these show considerable potential for aphid control, field trials employing the single and double constructs showed no reduction in aphids or increase in parasitism [62]. Since alkylpyrazine compounds may impact various insect species, heteroexpression of alkylpyrazine synthases in crops may enhance the resistance of the crop to diverse pest insects.

## Figures and Tables

**Table 1:** Insect species used in this study.

Species	Common name	Order	Family	Source
*Reticulitermes flavipes* Kollar	Eastern subterranean termite	Blattodae	Rhinotermitidae	Collected from Washington County, MS and maintained at BCPRU
*Coccinella novemnotata* Herbst	Nine-spotted lady beetle	Coleoptera	Coccinellidae	Reared at USDA-ARS, BCPRU, Stoneville, MS
*Neochetina eichhorniae* Warner	Water hyacinth weevil	Coleoptera	Curculionidae	Collected from Washington County, MS
*Aethina tumida* Murray	Small hive beetle	Coleoptera	Nitidulidae	Provided by Dr. Yucheng Zhu, USDA-ARS, SIMRU, Stoneville, MS
*Ades aegypti* Linnaeus	Yellow fever mosquito	Diptera	Culicidae	Reared at USDA-ARS, BCPRU, Stoneville, MS
*Musca domestica* Linnaeus	Housefly	Diptera	Muscidae	Reared at USDA-ARS, BCPRU, Stoneville, MS
*Aphis nerii* Boyer de Foscolombe	Oleander aphid	Hemiptera	Aphididae	Collected from Washington County, MS
*Rhopalosiphum padi* Linnaeus	Bird cherry oat aphid	Hemiptera	Aphididae	Collected from Washington County, MS
*Cimex lectularius* Linnaeus	Bed bug	Hemiptera	Cimicidae	Provided by Dr. Changlu Wang, Rutgers University, NJ
*Bagrada hilaris* Burmeister	Bagrada bug	Hemiptera	Pentatomidae	Reared at USDA-ARS, BCPRU, Stoneville, MS
*Nezara virdula* Linnaeus	Southern green stink bug	Hemiptera	Pentatomidae	Reared at USDA-ARS, BCPRU, Stoneville, MS
*Apis mellifera* Linnaeus	Honeybee	Hymenoptera	Apidae	Provided by Dr. Yucheng Zhu, USDA-ARS, Stoneville, MS
*Solenopsis invicta* Buren	Red imported fire ant	Hymenoptera	Formicidae	Field collected from Washington County, MS and maintained at BCPRU
*Solenopsis richteri* Forel	Black imported fire ant	Hymenoptera	Formicidae	Collected from Tunica County, MS and maintained at BCPRU
*S. invicta* X *S. richteri*	Hybrid imported fire ant	Hymenoptera	Formicidae	Collected from Washington County, MS and maintained at BCPRU
*Aphaenogaster picea* Wheeler	None	Hymenoptera	Formicidae	Collected from Washington County, MS and maintained at BCPRU
*Monomorium minimum* Buckley	Little black ant	Hymenoptera	Formicidae	Collected from Washington County, MS and maintained at BCPRU
*Monomorium pharaonic* Linnaeus	Pharaoh ant	Hymenoptera	Formicidae	Provided by Dr. Grzegorz A. Buczkowski, Purdue University, IN
*Nylanderia fulva* Mayr	Tawny crazy ant	Hymenoptera	Formicidae	Collected from Jackson County, MS
